# Polymeric Immunoglobulin Receptor Mediates Immune Excretion of Mucosal IgM–Antigen Complexes Across Intestinal Epithelium in Flounder (*Paralichthys olivaceus*)

**DOI:** 10.3389/fimmu.2018.01562

**Published:** 2018-07-19

**Authors:** Xiuzhen Sheng, Xiaoyu Qian, Xiaoqian Tang, Jing Xing, Wenbin Zhan

**Affiliations:** ^1^Laboratory of Pathology and Immunology of Aquatic Animals, KLMME, Ocean University of China, Qingdao, China; ^2^Laboratory for Marine Fisheries Science and Food Production Processes, Qingdao National Laboratory for Marine Science and Technology, Qingdao, China

**Keywords:** polymeric immunoglobulin receptor, IgM–antigen complex, gut-associated lymphoid tissue, immune excretion, flounder (*Paralichthys olivaceus*), transcytosis, mucosal immunity

## Abstract

Polymeric immunoglobulin receptor (pIgR) is one important player of mucosal defenses, but very little is known on pIgR-mediated immune excretion of the antigens that penetrate mucosal surface in fish. Previously, we cloned the pIgR of flounder (*Paralichthys olivaceus*) and developed anti-pIgR antibody. In this study, the flounders were immunized intraperitoneally with the chicken ovalbumin (OVA) and the control protein bovine serum albumin (BSA) to elicit mucosal IgM antibody and pIgR response, and then challenged with OVA *via* caudal vein injection after the immunized OVA was absent from fish body at the fourth week after immunization. After OVA challenge, strong OVA-positive fluorescence signals were observed in lamina propria (LP) submucosa and epithelial cells of the hindgut at 30 min, increased proceeding toward the distal portion of intestinal folds, reached a peak at 2–3 h, and then weakened and disappeared at 12 h, indicating that the OVA rapidly diffused from bloodstream into LP submucosa and excreted across intestinal epithelium. Whereas in BSA-immunized and OVA-challenged control fish, the OVA was detected in LP submucosa but not in intestinal epithelium due to the lack of OVA-specific antibody. Accordingly, in intestinal epithelium, the transepithelial transport of OVA was confirmed by immunogold electron microscopy, and co-localization of OVA, IgM, and pIgR was illuminated by multiple-label immunofluorescence confocal microscopy and analyzed using Image J software. Furthermore, in gut mucus but not in serum, an ~800-kDa protein band showed IgM-positive, OVA-positive, and pIgR-positive simultaneously, and the OVA, together with IgM and secretory component (SC) of pIgR, could be immunoprecipitated by anti-OVA antibody, demonstrating the existence of SC–polymeric IgM–OVA complexes. All these results collectively revealed that the pIgR could transport mucosal IgM–OVA complexes from LP across intestinal epithelium into gut mucus *via* the transcytosis in flounder. These new findings provided direct evidences for pIgR-mediated immune excretion of IgM–antigen complexes, and better understanding the role of pIgR in mucosal immunity in teleost fish.

## Introduction

Mucosal immunity is an important component of immune system, in which epithelial cells and lymphocytes function cooperatively. Mucosal surfaces, in fish including those of the gastrointestinal tract, skin, gills, and the olfactory organ, are in close encounter with multitudes of antigens and most infectious agents enter the host through mucosal membranes, so the mucosal barriers in aquatic animals are far more important than in their terrestrial counterparts (1–3). The polymeric immunoglobulins (pIgs) are the main players of mucosal defenses, and the unique structure of immunoglobulins (Igs) allows for simultaneous binding of antigen *via* the N-terminal variable regions of the Ig heavy and light chains and execution of effector functions *via* the C-terminal constant (Fc) region of the Ig heavy chains (4). To exert its protective effect, pIgs produced by local plasma cells in the lamina propria (LP) must be transported across epithelial cells to the luminal side, this process is termed transcytosis and mediated by polymeric immunoglobulin receptor (pIgR), another key component in mucosal defenses. The transport of pIgs by pIgR to the intestinal lumen is essential for protecting the host from invading pathogens and maintaining mucosal homeostasis in mammal (5), but in fish, very little is known on the role of pIgR in intestinal homeostasis, and studies of the mucosal barrier in the gastrointestinal tract of fish are in their infancy (1).

In mammals, the pIgR, which is expressed by the mucosal epithelia, ensures efficient secretion of polymeric IgA and IgM at mucosal surfaces and plays a pivotal role in mucosal immune protection, and secretory IgA (SIgA) is the principal Ig on mammalian mucosal surfaces. The pIgR mediates transport of pIgs across the glandular and mucosal epithelial cells, at the apical surface, the extracellular ligand-binding region of pIgR, known as secretory component (SC), is proteolytically cleaved off and released as a component of secretory Igs (SIgs) or in free form into external secretions functioning as an innate immune factor (1), these has expanded our view of the immunobiology of pIgR that bridges innate and adaptive immune defense. Actually, the functions of mammalian pIgR, well beyond transport of SIgs and free SC, also include that: (π) immune excretion of SIgA immune complexes; (θ) intracellular neutralization of invading pathogens; and (ρ) immune exclusion, i.e., pathogen neutralization within the lumen (6). However, data about these functions of fish pIgR are very limited. IgM is a predominant isotype in teleost body fluids, responding to pathogenic challenge in systemic and mucosal compartments of teleost fish (7), while IgT is recently found as a mucosal epithelial Ig to play a role in gut, skin, gill, and nasopharynx mucosal immunity in rainbow trout (*Oncorhynchus mykiss*) (8–11). Teleost pIgR has been shown to bind IgM in fugu (*Takifugu rubripes*), carp (*Cyprinus carpio*), orange-spotted grouper (*Epinephelus coioides*), and flounder (*Paralichthys olivaceus*) (9, 12–15), and bind IgT in rainbow trout and dojo loach (*Misgurnus anguillicaudatus*) (8–10, 12, 14–16), but no direct evidence for pIgR-mediated transepithelial transcytosis of SIgs is described so far. In rainbow trout, IgT, IgM, and IgD are reported to coat the microbiota in the skin, gut, and nasal mucus, suggesting mucosal Igs can mediate immune exclusion in the teleost fish (8–11). However, there is no reference about immune excretion of SIg-containing immune complexes reported in fish yet.

As one of four mucosal-associated lymphoid tissues in teleost fish, the gut-associated lymphoid tissue plays an important role in mucosal defense, since most pathogens gain access to the host through the gastrointestinal tract (17). In a number of teleost fish, it is well established that the second segment of the mid-intestine, also referred to as the hindgut, has special function in antigen uptake and probably is of immunological importance (17–19). In this study, to clarify the excretory function of fish pIgR, the pIgR-mediated immune excretion of mucosal IgM–antigen complexes, from lamina apropria across the epithelium into apical secretions, was investigated. The flounders were intraperitoneally immunized with the chicken ovalbumin (OVA) and the control protein bovine serum albumin (BSA) to elicit mucosal IgM antibody and pIgR response and thereafter a subsequent challenge with OVA antigen was given *via* caudal vein injection at the fourth week after immunization, in the hindgut of flounder, the OVA variation was analyzed by indirect immunofluorescence, the co-localization of OVA, IgM, and pIgR was determined by multiple-label immunofluorescence confocal microscopy and analyzed using Image J software, the transepithelial transport of the OVA in intestinal cells was illuminated by immunogold electron microscopy (IEM). Furthermore, the SC–IgM–OVA complexes excreted in gut mucus were confirmed by western blotting and co-immunoprecipitation assay.

## Materials and Methods

### Ethics Statement

This study was carried out in strict accordance with the recommendations of the Guidelines for the Use of Experimental Animals of Ocean University of China. The protocols for animal care and handling used in this study were approved by the Committee on the Ethics of Animal Experiments of Ocean University of China. Fish were anesthetized with ethyl 3-amino-benzoatemethanesulfonic acid (MS222) before sacrificing and handling.

### Fish and Antibodies

Totally 720 healthy flounders with length of 15–17 cm were obtained from a fish farm in Rizhao, Shandong province of China, where the fish were cultured in relatively strict conditions and the majority of fish had similar size and weight. During the experiment, fish were maintained in a temperature-controlled aquarium at 21 ± 1°C which was the optimal temperature to induce the most effective immune response of flounder (20), with continues aeration and recirculating water treated by UV and bio-filters, and fed daily with commercial dry food pellets. After acclimated to the laboratory setting for 7 days, the fish were checked by standard microscopical and bacteriological examination, and then subjected to immunization.

Mouse monoclonal antibody (MAb) 2D8 against flounder serum IgM which could recognize the heavy chain of serum and mucus IgM (21), and mouse polyclonal antibodies against flounder recombinant pIgR (15), were produced previously by our laboratory. Rabbit anti-OVA antibody was purchased from Sigma-Aldrich Productions GmbH (Steinheim am Albuch, Germany).

### Immunization and Challenge Strategy

The OVA (with a molecular weight of 43 kDa) (Sigma, V grade) and the control protein BSA (Sigma), both at a concentration of 10 mg/ml, were blended with Freund’s complete adjuvant (1:1, v/v) to a final concentration of 5 mg/ml, respectively, and used to immunize the flounders to stimulate a mucosal antibody response. The flounders were randomly divided into three groups, with three replicate tanks in each group, then intraperitoneally injected with 50 μl OVA mixture for group 1 and group 3, and equivalent volume of BSA mixture for group 2. On the fourth week after initiating immunization, the fish were challenged with OVA antigen (without adjuvant). Specifically, immunized fish in group 1 and group 2 were injected with 100 μl OVA at a concentration of 10 mg/ml *via* caudal vein, while those in group 3 were injected with equal volume of sterile phosphate-buffered saline (PBS, pH 7.2) as a control in which the fish were immunized but not challenged.

### Tissue Preparation and Serum/Gut Mucus Collection

Six flounders in each group were randomly sampled for the collection of fish hindgut on the fourth week after immunization, and at 30 min, 1 h, 1.5 h, 2 h, 3 h, 6 h, 9 h, and 12 h after the immunized fish were challenged respectively. Subsequently, the samples were treated for the preparation of cryostat and ultrathin sections. More specifically, the hindguts were immersed in tissue freezing medium (OCT, JUNG) immediately and frozen in −80°C, thereafter, the 5-μm-thick sections were cut and fixed with cold acetone for 10 min and stored at −20°C prior to use after air-dried in a fume cupboard. For ultrathin sections, the hindguts sampled at 2 and 9 h after challenge were fixed with 2% glutaraldehyde in PBS for 24 h, post-fixed with 1% osmium tetroxide after three washes with PBS, and then dehydrated with a graded ethanol series and embedded in Epon 812 (22). Ultrathin sections (50–70 nm thick) were made with ultramicrotome, and picked up onto nickel grids for IEM.

For serum and gut mucus collection, 15 fish in each group (5 fish from each tank) were randomly sampled at week 4 after initiating immunization, and at 3, 6, 12, 24, 48, and 96 h after challenge. The blood was drawn from the caudal vein of the fish before the tissue and mucus were collected. The blood was kept at 4°C overnight to allow clotting, and the serum was obtained by centrifugation at 3,000 × *g* for 10 min and then stored at −80°C before use. The mucus was collected according to the method by Zhang et al. (8) with minor modification. Briefly, following blood drawing completely, the fish were dissected to collect the hindgut which was then opened longitudinally, and 0.5 ml of protease inhibitor buffer (1× PBS, containing 1 mM phenylmethylsulfonyl fluoride and 0.5% BSA, pH 7.2) was added onto its surface. Approximate same volume of mucosal fluid from each fish was gently scraped from the inner surface of the gut and transferred to a centrifuge tube, followed by vigorous vortexing and centrifuging at 12,000 × *g* for 10 min. The resulting supernatant containing gut mucus was harvested, filtered with 0.45 μm syringe filter and then with Amicon Ultra-4 Centrifugal Filter Devices at 4,000 × *g* until the liquid volume was concentrated about 10-folds, with the same volume ratio in all samples. The upper liquid was thereafter collected and centrifuged at 12,000 × *g* for 10 min, and the supernatant was gathered and stored immediately at −80°C until use for electrophoresis assay and co-immunoprecipitation assay.

### Indirect Immunofluorescence Assay

To confirm the induction of mucosal antibody response and OVA absence in the intestinal mucosa on the fourth week after initiating immunization, the cryostat sections of flounder hindgut were utilized for IgM, pIgR, and OVA staining. Following washed in PBST (PBS containing 0.5% Tween 20) for 10 min, the sections were incubated with mouse anti-IgM MAb 2D8 (1:1,000), mouse anti-pIgR (1:1,000), and rabbit anti-OVA (1:3,000, Sigma) antibodies for 1 h at 37°C in a moisture chamber. For negative controls, incubations with myeloma culture supernatant instead of anti-IgM MAb 2D8, non-immune mouse and rabbit serum instead of mouse anti-pIgR and rabbit anti-OVA antibody, respectively, were conducted. After three washes with PBST, the sections were thereafter incubated with Alexa 488 conjugated goat anti-mouse IgG (Thermo Fisher Scientific, 1:1,000), Cy3 conjugated goat anti-mouse IgG (Sigma, 1:400), and Cy3 conjugated goat anti-rabbit IgG (Sigma, 1:400) for 45 min at 37°C in a moisture chamber. After washed again, the sections were stained with DAPI to visualize the nucleus for 10 min at room temperature and observed by fluorescence microscope.

To observe the variation of OVA in the intestinal mucosa, the cryostat sections of the hindgut, sampled at 30 min, 1 h, 1.5 h, 2 h, 3 h, 6 h, 9 h, and 12 h after OVA challenge, were used for OVA detection. Following preincubated with 3% BSA at room temperature for 1 h to block non-specific antibody binding, the sections were incubated with rabbit anti-OVA antibody (Sigma, 1:3,000) diluted in PBS overnight at 4°C, and then Cy3 conjugated goat anti-rabbit IgG (sigma, 1:400) for 45 min at 37°C. Subsequently, the sections were washed three times with PBST, stained with DAPI (Thermo Fisher Scientific, 1:1,000) for 10 min at room temperature, and detected by fluorescence microscope following washed for 4 min in double distilled water (DDW). For negative control, non-immune rabbit serum instead of rabbit anti-OVA antibody was used.

### Immunogold Electron Microscopy

The ultrathin sections, prepared from the hindgut at 2 and 9 h after OVA challenge, were washed with DDW for 5 min, and incubated with 1% H_2_O_2_ for 10 min. Following three washes in PBS, the sections were treated with 3% BSA for 1 h at 37°C and incubated with rabbit anti-OVA antibody for 1 h at 37°C. Non-immune rabbit serum instead of rabbit anti-OVA antibody served as negative control. After washed thrice in PBS, the sections were incubated with 3% BSA (dissolved in PBS, pH 8.3) for 10 min to provide an alkaline circumstance for the binding of colloid gold (23), then incubated with the goat-anti-rabbit IgG conjugated with 10 nm colloid gold (Sigma, 1:100) for 30 min at 37°C to trace the location of OVA in intestinal mucosa of flounder. Finally, the sections, without staining with uranyl acetate and lead citrate, were washed thoroughly with PBST and then DDW for three times, and observed with a transmission electron microscope.

### Multiple Immunofluorescence Labeling and Laser Scanning Confocal Microscopy

To explore if OVA antigen was excreted through the mucosal epithelium in the form of immune complexes *via* the pIgR, the IgM, pIgR, and OVA were simultaneously stained in the same slides of the hindgut from group 1 using mouse anti-IgM MAb 2D8, mouse anti-pIgR, and rabbit anti-OVA antibodies as probes. The specific procedures were as follows: frozen sections, prepared from the hindgut at 2 h after OVA challenge, were washed in PBST for 10 min, incubated with mouse anti-pIgR antibody (1:1,000) for 1 h at 37°C in a moisture box. After three washes with PBST, the sections were incubated with Alexa 647 conjugated goat anti-mouse IgG (Thermo Fisher Scientific, 1:1,000) for 1 h at 37°C in the dark. Following three washes, the sections were incubated with mouse anti-IgM MAb 2D8 (1:1,000) and rabbit anti-OVA antibody (Sigma, 1:3,000) mixtures (1:1, v/v) for 1 h at 37°C and washed again, and then incubated with Alexa 488 conjugated goat anti-mouse IgG (Thermo Fisher Scientific, 1:1,000) and Cy3 conjugated goat anti-rabbit IgG (Sigma, 1:400) mixtures (1:1, v/v) in 37°C for 45 min. After the redundant antibodies were washed off, the sections were stained with DAPI (Thermo Fisher Scientific, 1:1,000) for 10 min at room temperature to visualize the nucleus, and then detected by laser scanning confocal microscope. For negative controls, non-immune mouse serum instead of mouse anti-flounder pIgR, and myeloma culture supernatant paired non-immune rabbit serum (1:1, v/v) instead of mouse anti-IgM MAb 2D8 and rabbit anti-OVA antibody mixtures, were used. The quantitative image analysis for co-localization was performed using the image analysis software Image J, a public domain Java image processing program.

### Western Blotting and Co-Immunoprecipitation Assay

To investigate the relationship of OVA, pIgR, and IgM, serum and gut mucus samples were resolved on 3–20% Native-PAGE Ready Gel (Bio-Rad) under non-reducing conditions in duplicate, one was stained with Coomassie blue R-250, the other was transferred onto PVDF membrane (Millipore, USA). The membrane for western blotting was blocked with 3% BSA in PBS for 1 h at 37°C, followed by incubation with mouse anti-IgM MAb 2D8, mouse anti-pIgR, and rabbit anti-OVA antibody as the primary antibody, and AP-conjugated goat-anti-mouse and goat-anti-rabbit IgG (Sigma) diluted 1:4,000 in PBS as the secondary antibody for 1 h at 37°C. Finally, the bands were stained with freshly prepared substrate solution (100 mM NaCl, 100 mM Tris, and 5 mM MgCl_2_, pH 9.5) containing nitroblue tetrazolium chloride (NBT, Sigma) and 5-bromo-4-chloro-3-indolylphosphate toluidine salt (BCIP, Sigma) for 5 min, and stopped by washing with distilled water.

To further confirm that pIgR associated with OVA–IgM complexes in gut mucus, a co-immunoprecipitation experiment was performed using rabbit anti-OVA antibody to incubate with gut mucus of flounder. The 50-μl protein G agarose particles were reacted with 5 μl rabbit anti-OVA antibody in 1 ml PBS for 4 h at room temperature, centrifuged at 1,000 × *g* for 10 min, followed by discarding the supernatant and adding 1 ml PBS for three washing. Subsequently, 1 ml gut mucus up-mentioned was added and reacted overnight at 4°C, and then washed three times as before. Non-immune rabbit serum instead of rabbit anti-OVA antibody, or protein G agarose particles directly reacting with gut mucus from group 1, served as negative controls. Subsequently, the sediment was subjected to SDS-PAGE Ready Gel in duplicate, one was applied for staining with Coomassie blue R-250, and another was for western blotting analysis as described above by using mouse anti-IgM MAb 2D8 and mouse anti-pIgR antibody as primary antibodies and goat anti-mouse IgG as secondary antibodies, respectively.

### Statistical Analysis

The statistic *p* value was analyzed with one-way analysis of variance (SPSS Statistics, version 19, IBM), and the results were expressed as mean SEM. The significance level was defined as *p* < 0.05.

## Results

### OVA Immunization Elicited Mucosal IgM Antibody and pIgR Response

**Figure 1:**
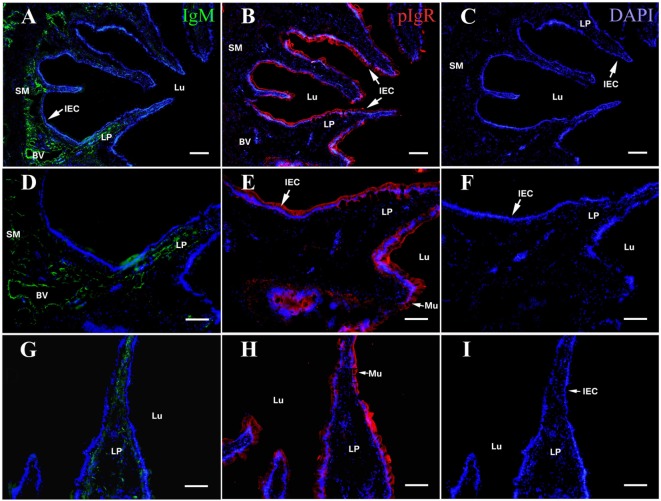
Indirect immunofluorescence of ovalbumin (OVA), pIgR, and IgM on the fourth week after initiating immunization of healthy flounder. **(A)** IgM was detected in lamina propria (LP) sunbmucosa of the fish hindgut of group 1 using anti-IgM monoclonal antibody 2D8. **(B)** The pIgR was distributed in mucosal epithelial cells of the fish hindgut of group 1. **(C)** No OVA was seen in the hindgut in group 1. **(D)** Higher magnification of **(A)**; **(E)** higher magnifications of **(B)**; **(F)** higher magnifications of **(C)**. **(G)** IgM staining in LP submucosa of the fish hindgut of group 2. **(H)** pIgR was distributed in mucosal epithelial cells of the fish hindgut of group 2. **(I)** No OVA was seen in the fish hindgut of group 2. Bar was 100 μm for **(A–C)** and 60 μm for **(D–I)**.

On the fourth week after the flounders were immunized, the distribution of OVA, IgM, and pIgR in hindgut was detected using rabbit anti-OVA antibody, mouse anti-IgM MAb 2D8, and rabbit anti-pIgR antibody as probes, respectively, and similar results were obtained in all three immunized groups. Evident IgM-positive green fluorescence was observed in LP submucosa, especially around the blood vessel (BV) walls (Figures 1A,D,G), and the pIgR-positive red fluorescence was localized in epithelial cell layer and in mucus lining (Figures 1B,E,H). However, there was no obvious OVA-positive red fluorescence was observed in the hindgut, showing that OVA disappeared in fish body at this time (Figures 1C,F,I). No positive fluorescence was detected in all negative control staining (data not shown). These results revealed that the immunized fish on the fourth week could be used to challenge OVA antigen for subsequent experiments.

### Mucosal IgM-Mediated Excretory of OVA Across Intestinal Epithelium

**Figure 2:**
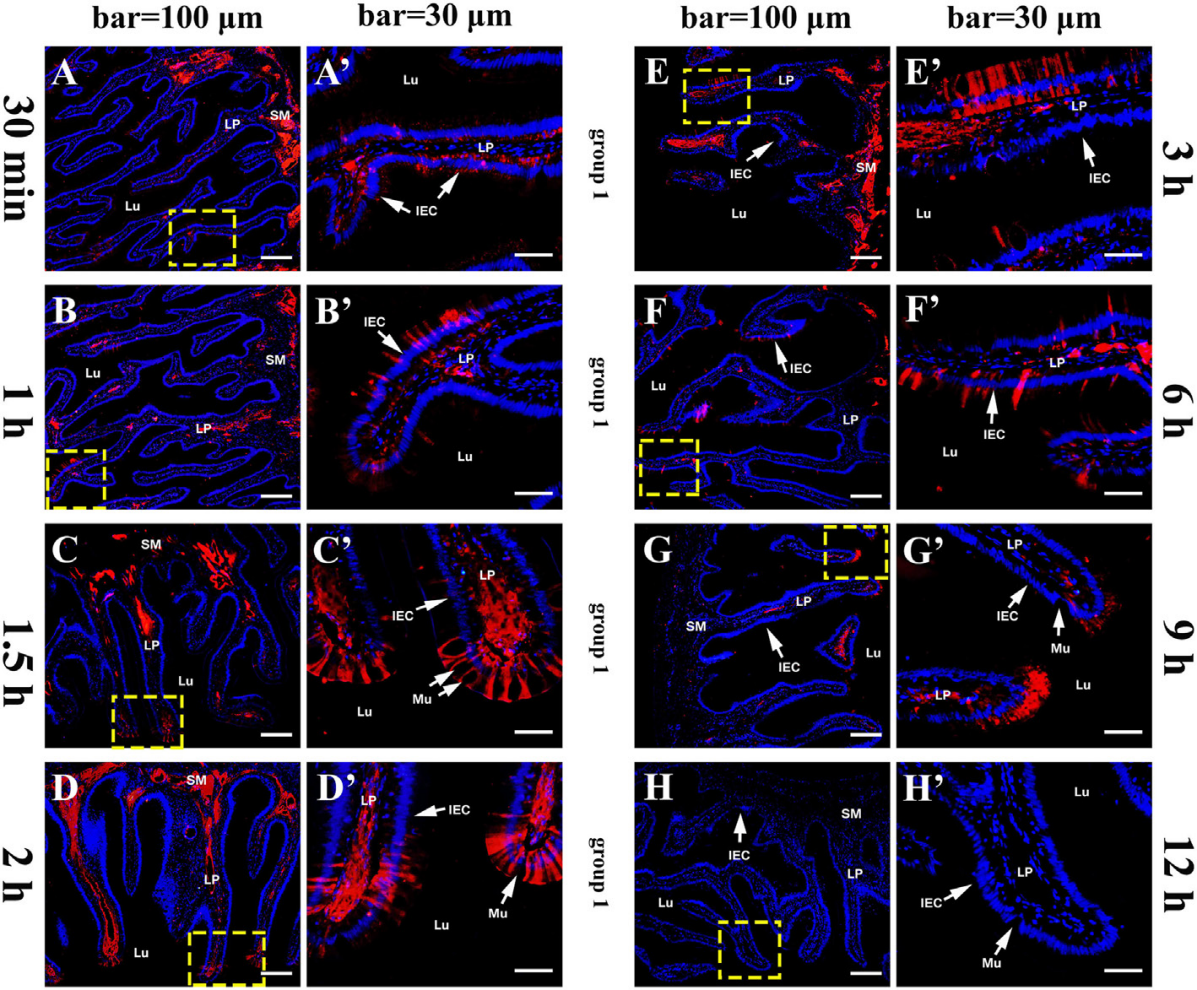
Tissue distribution of OVA in flounder hindgut of group 1 by indirect immunofluorescence assay at different time points after challenge. **(A–H)** The distribution of OVA at 30 min, 1 h, 1.5 h, 2 h, 3 h, 6 h, 9 h, and 12 h after challenge, respectively. Positive red fluoresce representing OVA was mainly located in LP submucosa and epithelial cells, accumulated with time and reached a peak at 2–3 h, then reduced from 6 h and disappeared at 12 h. Bar = 100 μm. **(A′–H′)**; the higher magnification view of the insert area (yellow box) in **(A–H)**, respectively, bar = 30 μm. The cell nuclei were counterstained in blue with DAPI. Abbreviations: LP, lamina propria; Lu, lumen; IECs, intestinal epithelial cells; Mu, mucus cells; OVA, ovalbumin.

**Figure 3:**
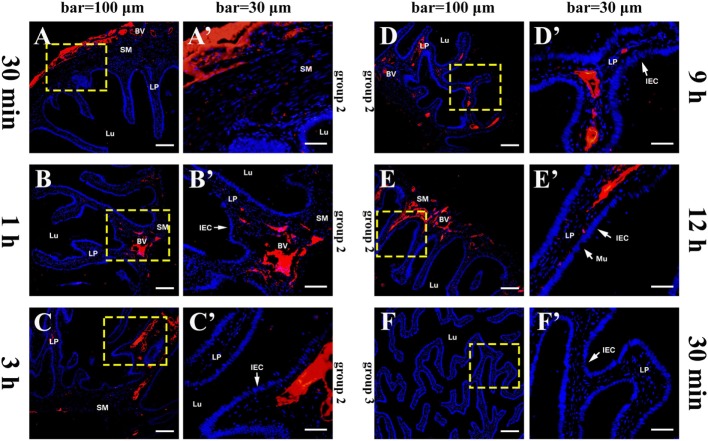
Tissue distribution of OVA in flounder hindgut of group 2 and group 3 by indirect immunofluorescence assay after challenge. **(A–E)** The distribution of OVA in group 2 at 30 min, 1 h, 3 h, 9 h, and 12 h after challenge, respectively, bar = 100 μm. Positive red fluoresce representing OVA was only detected in LP submucosa but not in the epithelial cells, and did not disappear at 12 h, showing that OVA could not be transported across epithelial cells from LP to lumen. **(A′–E′)** The higher magnification view of the insert area (yellow box) in **(A–E)**, respectively, bar = 30 μm. **(F,F′)** The results of group 3 at 30 min after challenge, showing no OVA-positive signals in the hindgut. Abbreviations: LP, lamina propria; Lu, lumen; IECs, intestinal epithelial cells; Mu, mucus cells; BV, blood vessel; OVA, ovalbumin.

The histological structure of flounder hindgut exhibited simple columnar epithelium with goblet cells (mucus-secreting cells) and underlying LP in intestinal mucosa which was branched and folded, but no obvious intestinal crypts at the fold base. The absence of a muscularis mucosa prevented the distinct separation of LP from the submucosa, and the LP submucosa contained a large number of BVs. After the immunized flounders in group 1 were challenged with OVA antigen *via* injection into caudal vein on the fourth week after immunization, at 30 min, a large of OVA-positive red fluorescence was observed in LP submucosa beneath the intestinal folds where the vascular network was surrounded by strong red fluorescence, and a few positive signals also presented in intestinal epithelial cells (IECs) and LP within intestinal folds (Figures 2A,A´). With the extension of time, the OVA was found to accumulate substantially in LP submucosa and mucosal epithelial layer, prominently proceeding toward the distal portion of intestinal folds where epithelial cells were present stronger OVA-positive (Figures 2B–D,B–D´), and the amount of OVA reached a peak at 2–3 h after challenge (Figures 2E,E´). Thereafter, the fluorescence intensity in LP submucosa begun to reduce at 6 h (Figures 2F,F´) and hardly observed at 9 h, but strong positive signals could be detected in epithelial layer and LP in apical position of intestinal folds at this time (Figures 2G,G´). At 12 h after challenge, the red fluorescence disappeared in both IECs and LP (Figures 2H,H´). In one control, the fish in group 2 were immunized with BSA and subsequently challenged with OVA, from 30 min to 12 h after challenge, positive red signals for OVA appeared and accumulated only in intestinal LP submucosa, but not in epithelial cells, and the distribution of the OVA were different from group 1 in that the staining was more localized around BVs (Figures 3A–E,A´–E´). In another control, the fish in group 3 were immunized with OVA and challenged with PBS, no OVA was detected all the time (Figures 3F,F´). No positive signals were observed in all negative control staining (data not shown). The results were consistent in all specimens.

### OVA Transepithelial Transport Evidenced in Intestinal Epithelium by IEM

**Figure 4:**
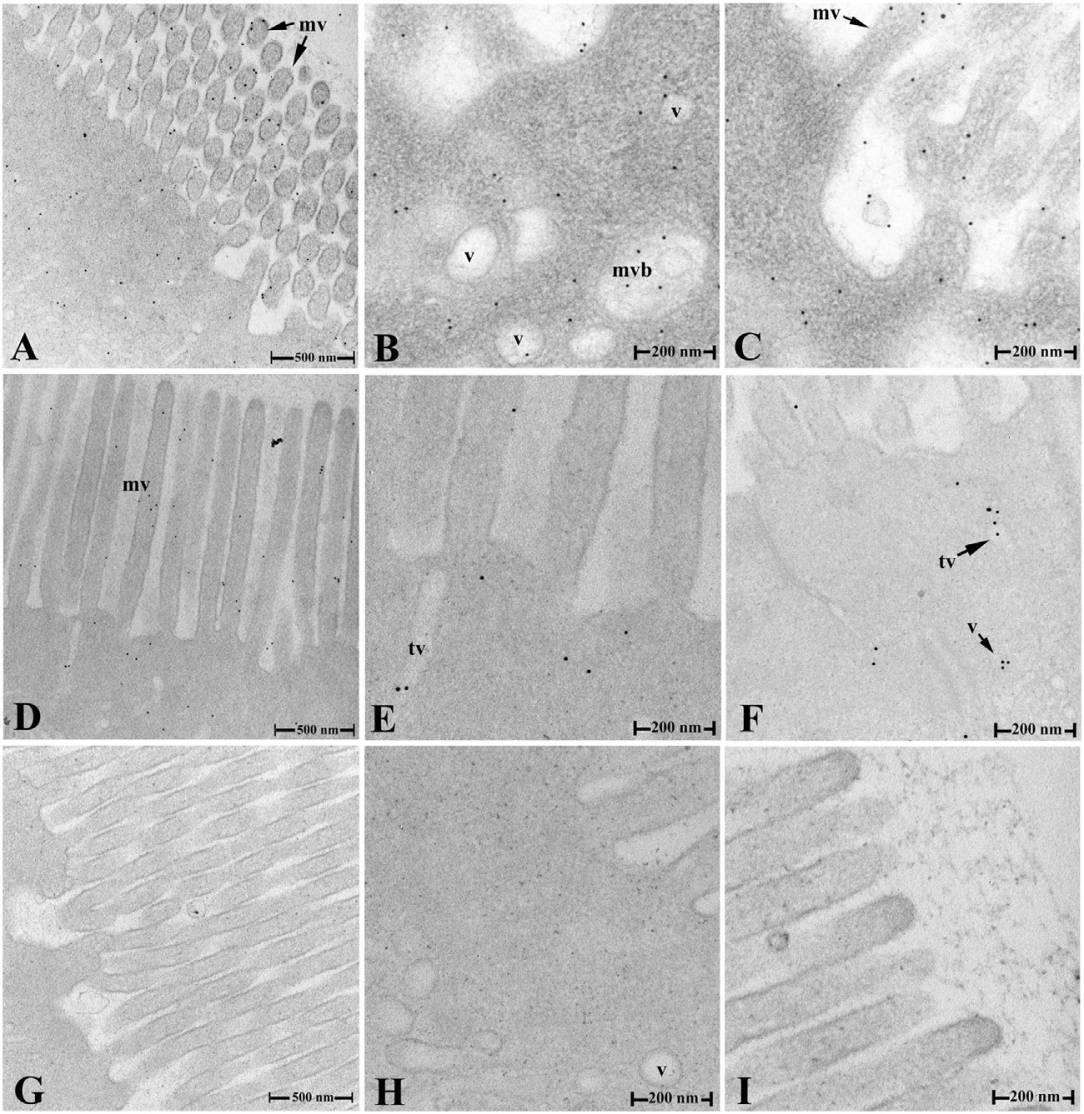
Transepithelial transport of the OVA evidenced by IEM. **(A–C)** The distribution of the OVA-positive gold particles in various sized vesicles, tubules, and multivesicular bodies in the cytoplasm, around the surface microvilli of IECs and in gut lumen in group 1 at 2 h after challenge. **(D–F)** The distribution of the OVA-positive gold particles in IEC of group 1 at 9 h after challenge. **(G–I)** No gold particles were detected in negative control staining **(G)** and IECs of hindgut in group 2 **(H,I)** at 2 h after challenge. Abbreviations: v, vesicles; tv, tubule vesicles; mvb, multivesicular bodies; mv, microvilli; IECs, intestinal epithelial cells; OVA, ovalbumin; IME, immunogold electron microscopy.

After OVA challenge, the ultrathin sections of fish hindgut were prepared at 2 and 9 h, and OVA in IECs was localized by immunogold transmission electron microscopy using rabbit anti-OVA antibody as probes. The results showed that, in fish of group 1, abundant gold particles were observed in the cytoplasm of IECs at 2 h, including in various sized vesicles (v), tubule vesicles (tv), and multivesicular bodies (mvb) within the cells, around surface microvilli (mv), and in the gut lumen (Figures 4A–C); while only a few gold particles were detected in the vesicles, tubules, and around microvilli at 9 h (Figures 4D–F). No gold particles were seen in IECs of group 2 (Figures 4H–I), group 3 (data not shown) and negative control (Figures 4,G).

### Co-Localization of the OVA, IgM, and pIgR in Intestinal Epithelium

**Figure 5:**
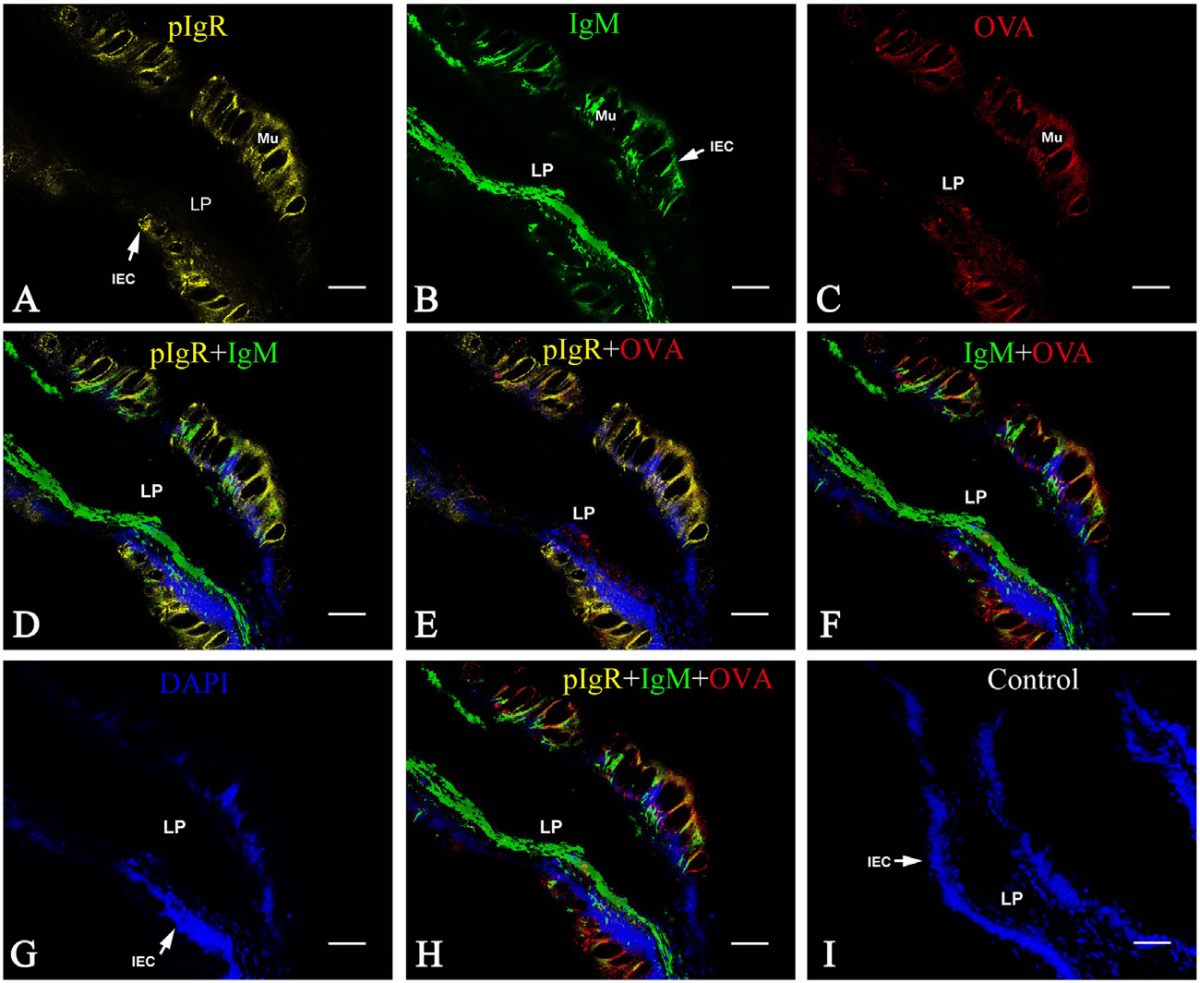
Co-localization of pIgR, IgM, and OVA in hindgut of flounder. Pictures from group 1 were shown as examples. The fish immunized intraperitoneally with OVA and subsequently challenged with OVA *via* caudal vein on the fourth week after immunization, and pIgR, IgM, and OVA in hindgut was stained at 2 h after challenge, presenting orange, green and red fluoresce respectively. The cell nucleus was counterstained in blue with DAPI. **(A,B,C)** Distribution of IgM, OVA, pIgR in hindgut at 2 h after challenge was illuminated, respectively. **(D–F)** The merged picture of **(A,B,G)** and **(A,C,G)** and (**B,C,G)**, respectively. **(G)** The cell nucleus of intestinal epithelium was stained blue by DAPI. **(H)** The merged picture of **(A–C,G)**; **(I)** Negative control. Bar = 25 μm. Abbreviations: LP, lamina propria submucosa; Lu, lumen; IECs, intestinal epithelial cells; Mu, mucus cells; BV, blood vessel; OVA, ovalbumin.

Multiple immunofluorescence staining was used to confirm if the OVA transport from LP across intestinal epithelium was in the form of pIgR–IgM–OVA complexes. Taking the samples of group 1 at 2 h after OVA challenge as an example, the results demonstrated that the pIgR-positive orange fluorescence signals were distributed in the epithelial layer (Figure 5A), IgM-positive green fluorescence signals were clustered in intestinal LP submucosa and epithelial cells (Figure 5B), and the OVA-positive red fluorescence signals were located mainly in the epithelial layer and some in LP submucosa (Figure 5C). From the merged figure, it was found that pIgR and IgM (Figure 5D), pIgR and OVA (Figure 5E), IgM and OVA (Figure 5F), as well as pIgR, IgM, and OVA (Figure 5H), could be co-localized in the epithelial cells, whereas in LP submucosa, the amount of IgM was more than that of OVA. Cell nuclei were counterstained in blue by DAPI (Figure 5G). No obvious fluorescence was observed in negative control (Figure 5I).

**Figure 6:**
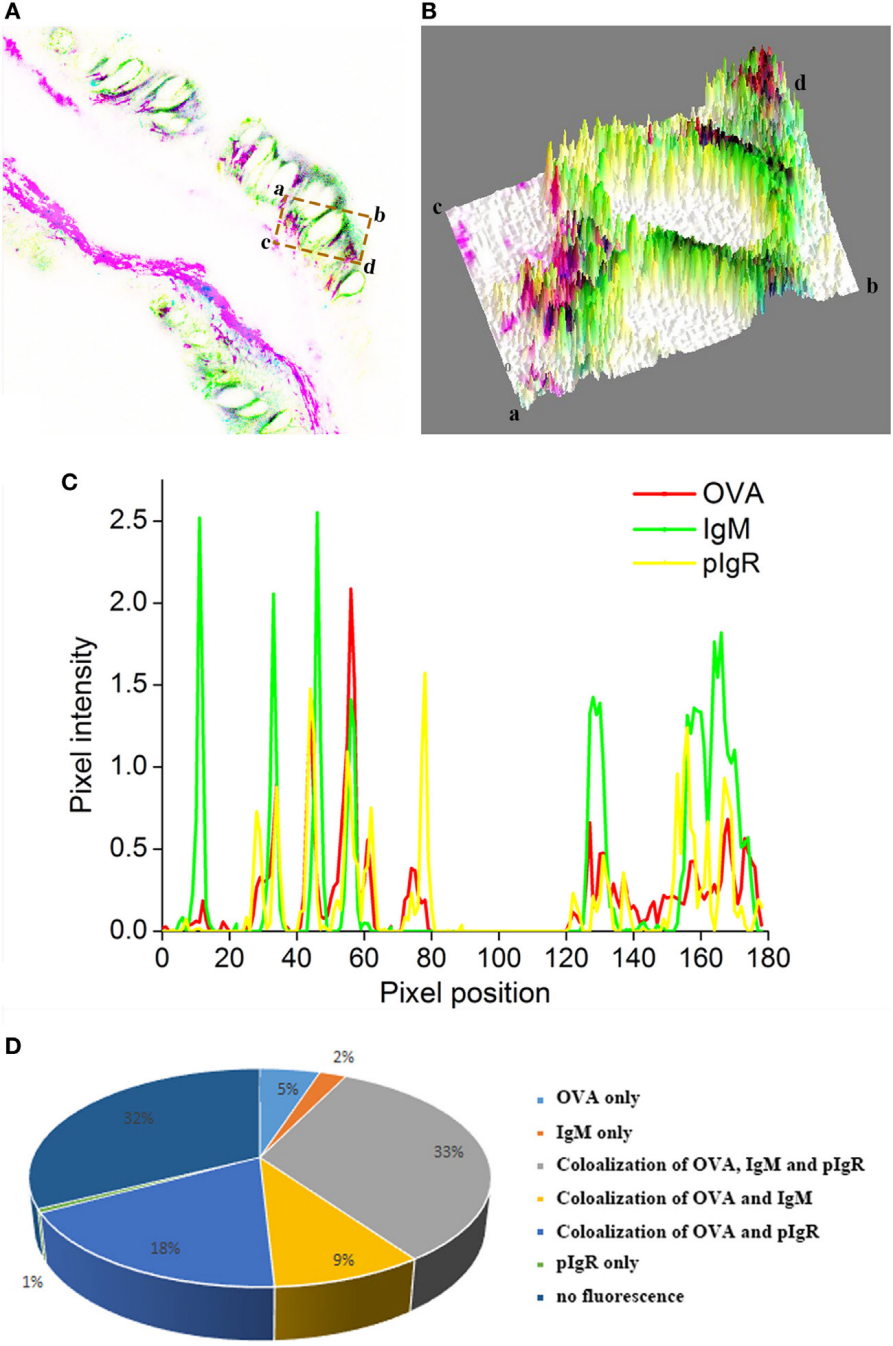
Quantitative image analysis for co-localization of ovalbumin (OVA), IgM, and pIgR in a selected area of intestinal epithelium using software Image J. **(A)** A new merged image of Figures 5A–C using blue, green, and red to represent pIgR, IgM, and OVA-positive signal, respectively. **(B)** The interactive 3D surface plot of the selected area (rectangle a-b-c-d) of **(A)**. **(C)** Analysis of co-localization of OVA, IgM, and pIgR according to the optical density of three fluorescence in the selected area. **(D)** A pie chart to show the percentage of OVA, IgM, and pIgR co-localization.

Quantitative image analysis for co-localization of OVA, IgM, and pIgR in intestinal epithelium was performed using Image J software. A new merged image of Figures 5A–C was generated using blue, green, and red to represent pIgR, IgM, and OVA-positive signal, respectively (Figure 6A), an area of epithelial layer in the merged image was selected (rectangle a-b-c-d), and the interactive 3D surface plot of the selected area was indicated in Figure 6B, pixels with positive signals for three probes were shown in black (Figures 6A,B), meaning the co-localization of OVA, IgM, and pIgR. The optical density of three fluorescence in the selected area was measured *via* software Image J, and the background color and noisy point were eliminated by automatically setting a threshold for each channel. The optical density was plotted and the overlapping pixels in abscissa axis were considered co-localized area of OVA, IgM, and pIgR (Figure 6C). The percentage values were displayed on a pie chart (Figure 6D), the results indicated that 179 pixels were obtained in the selected area, of which, 33% represented co-localization of OVA, IgM, and pIgR, 18% represented co-loalization of OVA and pIgR, and 9% represented co-localization of OVA and IgM, while 5, 2, and 1% were single OVA, IgM, and pIgR signal, respectively, and 32% of selected area had no fluorescence.

### Flounder pIgR Associated With IgM–OVA Immune Complexes in Gut Mucus

**Figure 7:**
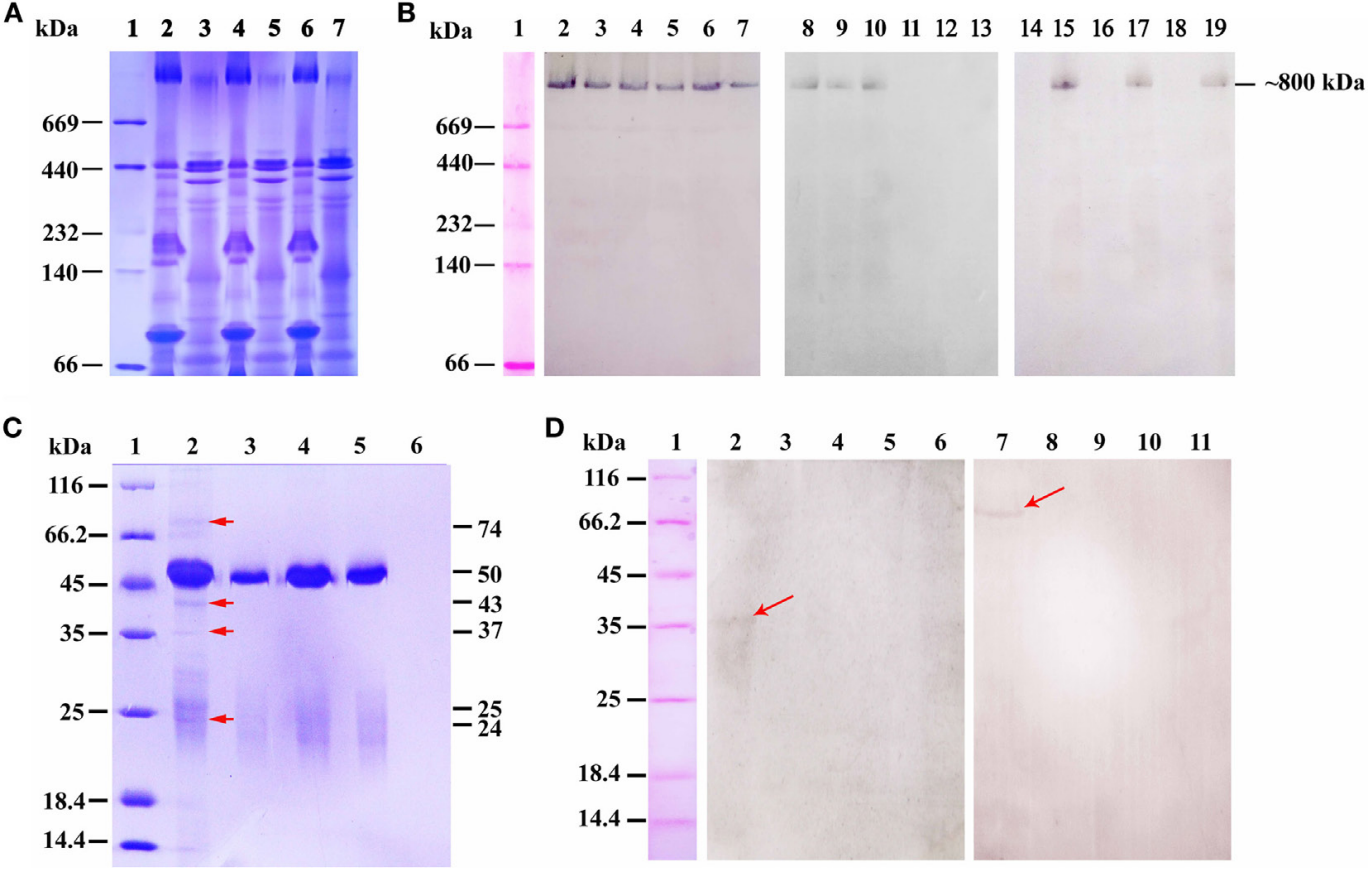
The pIgR associated IgM–ovalbumin (OVA) complexes in intestinal mucus of group 1 at 6 h after OVA challenge. **(A,B)** The Native-PAGE under non-reducing condition that stained by Coomassie blue and analyzed by western blot. **(A)** Lane 1: maker, lanes 2–7: native-PAGE result of serum and gut mucus of group 1 (2–3), group 2 (4–5), and group 3 (6–7), respectively. **(B)** Western blot result. Lane 1: maker, lanes 2–7: the samples of **(A)** reacted with anti-IgM monoclonal antibody (MAb) 2D8; lanes 8–13: the samples of **(A)** reacted with rabbit anti-OVA antibody; lanes 14–19: the samples of **(A)** reacted with mouse anti-pIgR antibody. **(C)** SDS-PAGE under reducing condition analyzed the samples from co-immunoprecipitation of gut mucus with rabbit anti-OVA antibody after challenge. Lane 1: maker; lane 2: protein G + rabbit anti-OVA antibody + gut mucus of group 1; lane 3: protein G + rabbit anti-OVA antibody + gut mucus of group 2; lane 4: protein G + rabbit anti-OVA antibody + gut mucus of group 3; lane 5: protein G + non-immune rabbit serum + gut mucus of group 1; lane 6: protein G + gut mucus of group 1. **(D)** Co-immunoprecipitation of gut mucus with anti-OVA antibody, followed by western blotting assay under reducing conditions. Lane 1: maker; lanes 2–6: Samples of **(C)** reacted with mouse anti-pIgR antibody; lanes 7–11: Samples of **(C)** reacted with anti-IgM MAb 2D8.

Under non-reducing conditions, the gut mucus and serum of the challenged fish were analyzed by native-PAGE, and a distinct band at a molecular weight of ~800 kDa was found in serum and mucus of all three groups after stained with Coomassie blue R-250 (Figure 7A). Western blotting assay revealed that the ~800 kDa protein band of the three groups could be specifically recognized by anti-IgM MAb 2D8 (Figure 7B, lanes 2–7); However, only the ~800 kDa protein band in serum and gut mucus of group 1, as well as in serum of group 2, could react with anti-OVA antibody (Figure 7B, lanes 8–13). Moreover, the ~800 kDa protein band in gut mucus, but not in serum, could reacted with anti-pIgR antibody in three groups (Figure 7B, lanes 14–19). All these results revealed that the pIgR–IgM–OVA complexes only existed in gut mucus of the fish in group 1.

To further confirm that the pIgR associated with IgM–OVA complexes, the gut mucus of the fish in three groups was purified and co-immunoprecipitation assay was performed with rabbit anti-OVA antibody, and SDS-PAGE results showed that, after Coomassie blue staining, distinct protein bands at 74, 50, 43, 37, 25, and 24 kDa were observed in gut mucus of group 1 (Figure 7C, lane 2), while the 50- and 25-kDa protein bands were also found in gut mucus of group 2 and 3 (Figure 7C, lanes 3 and 4), as well as the control using non-immune rabbit serum replacing of rabbit anti-OVA antibody (Figure 7C, lane 5), but no band was observed in the control that protein G directly reacted with gut mucus (Figure 7C, lane 6), indicating that the 50- and 25-kDa proteins were the heavy and light chain of rabbit IgG, respectively, whereas other proteins were in accordance with the molecular masses of the heavy chain of flounder IgM, OVA, extracellular portion of pIgR (SC), and light chain of flounder IgM, respectively. Thereafter, western blotting was carried out by using anti-IgM MAb 2D8 and anti-pIgR antibody as probes, and the results demonstrated that anti-IgM MAb 2D8 could recognize the 74-kDa protein (Figure 7D, lane 7), while anti-pIgR antibody could recognize the 37-kDa protein (Figure 7D, lane 2), revealing that the IgM and SC of pIgR in gut mucus together with the OVA could be immunoprecipitated by the anti-OVA antibody.

## Discussion

In mammals, reference indicates that there is a mucosal IgA-mediated excretory immune system, and SIgA can excrete antigens from the body by transporting them directly through mucosal epithelial cells with the help of pIgR (24). However, there are no data about whether a similar excretory immune system exists in teleost fish. Previously, we have cloned flounder pIgR and developed anti-pIgR antibody and found a SC-like molecule in skin mucus but not in serum of flounder (15). In this study, to stimulate a mucosal antibody response, healthy flounders were intraperitoneally immunized with the OVA, a common antigen with a strong immunogenicity, after IgM induction in the LP submucosa, pIgR synthesis in intestinal epithelium, and OVA absence in fish body were confirmed, the fish were challenged with a large dose of OVA *via* caudal vein injection to provide sufficient soluble IgM–OVA immune complexes in intestinal LP submucosa for their excretion through mucosal epithelium to be detectable morphologically. Using this *in vivo* model, the immune excretion of the challenged OVA antigens was investigated in flounder hindgut, and it was found that the OVA rapidly diffused from bloodstream into LP submucosa and excreted across the epithelial cells by pIgR in the form of pIgR–IgM–OVA complexes into gut mucus within 12 h, revealing that a mucosal IgM-mediated excretory immune system existed in flounder, in which the pIgR was essential for transporting IgM–antigen immune complexes across polarized epithelium from LP to the apical surface.

In mice, it is found that the immune complex is processed along the excretory pathway when the pIgA encounters the antigen within the underlying LP, and subsequent pIgR-mediated transcytosis leading to the release of antigen–SIgA complexes in intestinal crypts (24). Moreover, measles virus excretion is reported in Madin–Darby canine kidney cells which are stably transfected with cDNA encoding human pIgR, showing IgA transports virus into and through polarized epithelial cells (25). Whereas in teleost fish, the mechanism by which mucosal Igs containing complex gains access to the lumen across mucosal epithelium remains undefined. In this study, the flounders in group 1 were challenged with OVA *via* caudal vein after mucosal IgM and pIgR responses were induced by OVA immunization, and strong OVA-positive fluorescences were observed around vascular network in LP submucosa, and some in intestinal epithelium at 30 min, peaked at 2–3 h, then declined and disappeared at 12 h. All these results collectively revealed that the challenged OVA antigens diffused from bloodstream *via* the vascular network into the LP submucosa, and the immune system of flounder could response rapidly to the foreign antigens, and excreted them across the intestinal epithelium into gut mucus. On the contrary, in the BSA-immunized and OVA-challenged control fish of group 2, the OVA-positive fluorescences were present in LP submucosa and seemed to be more localized around the BV, but not in epithelium, from 30 min to 12 h, demonstrating that no OVA excretion occurred due to the lack of OVA-specific IgM. Combined these results, we could conclude that the free OVA antigens accessing the LP submucosa would immediately encounter OVA-specific IgM that had been induced to form OVA–IgM immune complexes, of course, some OVA binding to OVA-specific IgM in the circulation might also diffuse into the LP submucosa, and they thereafter were transported into gut mucus mediated by the pIgR, so the given OVA-specific IgM reacting with OVA was an important determinant of the immune excretion. Therefore, the immune excretory routes of foreign antigens in flounder were similar to the higher vertebrates (24) to a certain extent; however, the OVA–IgM immune complexes in LP submucosa of flounder hindgut were excreted across the epithelial cells of intestinal folds into gut lumen, predominately at apical position of the folds, rather than in intestinal crypts due to inexistence of the crypts at the fold base of flounder hindgut. Nevertheless, other alternative mechanism, such as phagocytic activity of macrophages and neutrophils, which played an important role in limiting the dissemination of infectious agents and were responsible for the eventual destruction of phagocytosed pathogens in teleost fish (26), might also contribute to the elimination of OVA in flounder in addition to pIg transport, further research was worth doing to clarify this.

The transcytosis mediated by pIgR have been studied a lot both *in vivo* (27) and *in vitro* (28, 29) in mammals, and SIgA is transported across epithelial cells into the lumen through an active basolateral-to-apical unidirectional process involving the pIgR (5). Compared with IgA transport mediated by pIgR, the FcRn-mediated IgG or immune complex transport exhibits bidirectionality (apical to basal and basal to apical direction) without proteolytic cleavage (30). In teleost fish, very little is known about the specific details of the process of transporting mucosal Igs or immune complexes *via* pIgR-mediated transcytosis. In this study, after the immunized flounders of group 1 were challenged with OVA, (1) the pIgR was still located in intestinal epithelium, but IgM was distributed in both LP submucosa and epithelium, rather than just in LP submucosa as before challenge; (2) IgM was co-localized with pIgR and OVA in intestinal epithelium; (3) a ~800 kDa protein band in gut mucus could react with anti-pIgR, anti-IgM, and anti-OVA antibodies, and anti-OVA antibody could immunoprecipitate the OVA along with IgM and SC of pIgR in gut mucus of group 1, but not group 2 and 3. All these results together revealed that the OVA, IgM, and pIgR existed in intestinal epithelium and gut mucus as a complex, and the pIgR transported polymeric IgM–OVA complexes from LP submucosa across intestinal epithelium through a basolateral-to-apical unidirectional process, and excreted into gut mucus in the form of SC–IgM–OVA complex, which provided direct evidences for pIgR-mediated transcytosis of IgM containing complexes *in vivo* in fish. During this process, IgM binding to OVA and then to SC of pIgR constituted a crucial step initiating the transcytosis, which served as a means for quickly removing antigens from mucous membranes, thereby minimizing the burden of immune complexes and achieving efficient protection of all three compartments of the epithelial barrier (LP, epithelium, and lumen) of fish, in this way, the excretory function of IgM *via* pIgR also could help to prevent diseases and play crucial role in defense against pathogens. Furthermore, the transepithelial transport of OVA in IECs was also confirmed by IEM, and distinct gold particles was found in various sized vesicles, tubule vesicles, multivesiculear bodies, around the surface microvilli of IECs and in gut lumen of flounder in group 1, but no gold particles were seen in IECs of group 2, group 3, and negative control, suggesting the gold particles in group 1 was specific staining. These results was consistent with the references that the proteins were sorted into transcytotic vesicles (28), and various vesicles, tubules and multivesicular bodies in epithelial cells involved in the process of IgA transport in mammals (31). On the other hand, quantitative image analysis for immunofluorescence data revealed that 33% of pixels in the selected area represented co-localization of OVA, IgM, and pIgR, suggesting the SC–IgM–OVA complexes formed in intestinal epithelium.

With regard to the ~800 kDa protein band which could react with anti-OVA antibody in serum but not in gut mucus in BSA-immunized and OVA-challenged control group 2, it might be because of the natural or non-specific IgM in serum that bound OVA antigen in blood, as reported in common carp that natural antibodies could react to OVA, BSA, or keyhole limpet hemocyanin (32), and injections with Freund’s complete adjuvant led to increased antibody activity in cod (*Gadus morhua* L.) (33). However, the baseline IgM expression prior to OVA/BSA application in untreated fish was not detected in this study, so further confirmation was required in flounder in the future. Natural antibodies are of low affinity and mostly of the IgM isotype. A relatively high level of natural antibodies is found in serum of normal individuals in cod, which reacts to a variety of haptenated antigens and bacterial membranes, providing an instant protection against pathogens of a broad specificity and participating in homeostasis (17, 34, 35). However, whether the natural antibody can also be transcytosed by pIgR is not clear to date. In this study, no OVA-positive band at ~800 kDa in gut mucus of group 2 was detected, suggesting no natural antibody-mediated OVA transport by pIgR occurred in absence of specific anti-OVA antibody. Furthermore, it is interesting that the naïve IgM was around 800 kDa as shown in serum of group 3 which just reacted with anti-IgM MAb, whereas the positive band in gut mucus and serum of group 1, which reacted with anti-IgM, anti-OVA, and anti-pIgR antibody, or with anti-IgM and anti-OVA antibody, respectively, possessed similar molecular mass, these complexes containing OVA and SC of pIgR in gut mucus, or OVA in serum should be much larger in theory, but that was not the case. In trout, mucosal IgM is found to be significantly less polymerized than serum IgM (36), and pIgR is suggested to participate in the selective removal of lightly cross-linked IgM from the circulation and delivery to the mucosa (37). In teleost, it is proposed that final disulfide polymerization of IgM does not occur until arrival at a late stage in the secretory process, in this model secretion itself may be the determining factor of the degree of complete disulfide crosslinking (38). Therefore, the assembly mechanism by which flounder polymeric IgM binds to pIgR and OVA for transcytosis and the structure of this complex worth more researches.

The research about mice deficient for pIgR revealed that this is the only receptor responsible for epithelial transport of IgA and IgM (39). As the predominant isotype in teleost body fluids, IgM play important role in systemic and mucosal compartments of teleost fish (7). Besides, the trout IgT is recently considered as a specialized mucosal epithelial Ig in fish and equivalent to the mammalian IgA (8–11, 15). We also find that flounder IgT mRNA level is upregulated after vaccination and infection with *Edwardsiella tarda*, higher in gill, skin, hindgut, liver, and stomach in immersion than in injection group, but no significant difference exist in spleen and head kidney between the two groups, indicating that flounder IgT may play a more important role in mucosal than systemic immunity (40); however, whether flounder IgT can bind the pIgR is not known because of the lack of anti-flounder IgT antibody, therefore, further studies about flounder pIgR-mediated excretion of IgT-containing immune complex are needed in the future.

In conclusion, the immune excretion of SIgM–OVA immune complexes across intestinal epithelium mediated by pIgR was investigated in flounder in this study. An *in vivo* experimental model was developed by intraperitoneal immunization with OVA to stimulate specific IgM antibody against OVA and pIgR response, and then a subsequent OVA challenge was given *via* caudal vein injection at the fourth week after immunization. The results revealed that a mucosal IgM-mediated excretory immune system existed in teleost fish, in which the pIgR transported polymeric IgM–antigen complexes from LP across intestinal epithelium by the transcytosis, through a basolateral-to-apical process, into gut mucus, thereby excluding antigen and immune complexes from the systemic circulation, and achieving efficient protection of all three compartments of the mucosal barrier (LP, epithelium, and lumen). To the best of our knowledge, this is the first report on pIgR-mediated immune excretion of IgM–antigen immune complexes in fish.

## Ethics Statement

This study was carried out in strict accordance with the recommendations of the Guidelines for the Use of Experimental Animals of Ocean University of China. The protocols for animal care and handling used in this study were approved by the Committee on the Ethics of Animal Experiments of Ocean University of China. Fish were anesthetized with ethyl 3-amino-benzoate-methanesulfonic acid (MS222) before sacrificing and handling.

## Author Contributions

XS designed the research, analyzed the data, and wrote the manuscript. XQ performed the experiments and helped with data analysis and manuscript writing. XT helped with most of the experiments. JX helped the reagent preparation and participated in data analysis. WZ designed the research and revised the manuscript.

## Conflict of Interest Statement

The authors declare that the research was conducted in the absence of any commercial or financial relationships that could be construed as a potential conflict of interest.
